# In Vitro Digestibility and Rheological Properties of Chinese Yam (*Dioscorea opposita* Thunb.) Starch Modified with Chlorogenic Acid

**DOI:** 10.3390/foods15203655

**Published:** 2026-10-15

**Authors:** Sandu Xie, Huiqing Chen, Liping Li, Ting Wang, Beini Wang, Hongliang Zeng, Yi Zhang, Baodong Zheng

**Affiliations:** 1 Fujian Provincial Key Laboratory of Quality Science and Processing Technology in Special Starch, Fujian Agriculture and Forestry University, Fuzhou 350002, China; 2 School of Life Sciences and Chemistry, Minnan Science and Technology College, Quanzhou 362332, China; 3 College of Food Science, Fujian Agriculture and Forestry University, Fuzhou 350002, China

**Keywords:** Chinese yam starch, chlorogenic acid, in vitro digestibility, rheological properties, structural characteristics

## Abstract

Chlorogenic acid (CA) has received extensive attention in the field of improving the properties of starch. At present, few studies have focused on the influences of chlorogenic acid (CA) addition levels on the physicochemical properties of Chinese yam (*Dioscorea opposita* Thunb.) starch (CYS). Therefore, the effects of varying chlorogenic acid (CA) concentrations (3%, 5%, and 7%) on the structural characteristics, in vitro digestibility, and rheological properties of Chinese yam starch (CYS) and its CA-incorporated complexes was investigated. The incorporation of CA disrupted the double-helix structure of starch, while promoting the formation of double- and single-helical structures in amylose, as revealed by Fourier-transform infrared spectroscopy and Raman spectroscopy, respectively. This led to decreased crystallinity and the formation of a V-type crystalline structure. The interactions between CA and CYS molecules facilitated the construction of a more compact network architecture within starch granules, thereby decreasing its in vitro digestibility and significantly increasing resistant starch content. Moreover, CA enhanced the short-range ordered structure of starch, restricted granule swelling, strengthened shear-thinning behavior, and reduced gel strength and viscosity. In summary, the complexation between Chinese yam starch and chlorogenic acid provides a promising strategy to tailor the rheological properties and in vitro digestibility of starch, providing a promising candidate substrate for fabricating low-digestibility starchy functional food formulations.

## 1. Introduction

The management of diet-related diseases, particularly metabolic diseases such as obesity, type II diabetes, and cardiovascular disease, is receiving increasing attention. Numerous studies have shown that these diseases are closely associated with the long-term consumption of foods with a high glycemic index (GI > 70) [1,2,3]. Starch is a carbohydrate-rich biopolymer that is widely used in food systems for its thickening, gelling, and stabilizing properties. Initially, starch in foods was assumed to be completely digested and absorbed, releasing glucose rapidly into the bloodstream and thereby causing a sharp rise in postprandial blood glucose levels, which was in turn thought to contribute to various metabolic disorders. As a result, foods with a low glycemic index are experiencing growing demand. However, as research on starch digestion progressed, researchers found that, in addition to easily digestible starch and slowly digestible starch, a small fraction of the starch resisted enzymatic hydrolysis [4,5]. Starch components that are not digested and absorbed in the small intestine of healthy humans are classified as resistant starch (RS). RS is fermented by gut microbiota to yield short-chain fatty acid metabolites (SCFAs), primarily acetate, propionate, and butyrate, which confer health benefits, including improved laxation, decreased serum cholesterol, and a reduced risk of diabetes [6,7]. Thus, RS is recognized as a potential prebiotic and plays an important role in metabolic regulation and colon health. Accordingly, increasing the RS content of starch-rich foods has been proposed as a promising nutritional intervention for patients with diet-related metabolic diseases. In addition to its functional properties, RS exhibits desirable physico-chemical properties and processing characteristics, making it useful in a variety of extruded and baked products, such as grain-based and low-moisture foods [7]. Based on their physical and chemical properties, RSs can be classified into five categories [8]: RS1, physically trapped starch, enclosed within intact cell walls; RS2, partially crystalline native starch granules; RS3, retrograded or crystalline starch; RS4, chemically modified starch; RS5, amylose–lipid complex starch [9]. Of these, amylose–lipid complex starch (RS5) represents a newly recognized type. Studies have shown that gut microbiota ferment RS5 to produce greater amounts of SCFAs than they do with RS2 or RS3 [10].

The interactions between starch and various ligands (e.g., alcohols, lipids, polyphenols, and flavor compounds) typically alter the short- and long-range molecular order of starch, consequently affecting important processing characteristics, including digestibility and rheology. Chlorogenic acid (CA), a typical polyphenol widely distributed in plant-based foods, exhibits proven antioxidant, anti-inflammatory and hypoglycemic bioactivities. Under specific conditions, CA and other polyphenols can interact with starch to form inclusion complexes, including single left-handed helices (V-type amylose) or non-covalent complexes stabilized mainly by hydrogen bonds, which can be influenced by high temperatures or strong external forces [6,11]. Moreover, starch–polyphenol V-type complexes have a crystalline arrangement similar to starch–lipid V-type complexes and are classified as type 5 RS (RS5), which can reduce starch digestibility. This inhibitory effect may result from the non-covalent interactions between starch and polyphenols, leading to the formation of insoluble complexes that hinder the enzymatic hydrolysis of the starch [12]. The development of functional foods has accelerated in recent years, with starch–polyphenol V-type complexes emerging as promising candidates due to their ability to reduce starch digestibility while increasing the content of resistant starch (RS5). Overall, starch–polyphenol V-type complexes show promise as a strategy for controlling postprandial hyperglycemia [13].

Starch is the main component of Chinese yams (*Dioscorea opposita* Thunb.), accounting for approximately 18–25% of their fresh weight. However, native Chinese yam starch exhibits undesirable properties during food processing, such as unstable paste consistency, low heat and shear resistance, syneresis during storage, and poor solubility [14]. Therefore, modification strategies are required to improve the processing characteristics of native Chinese yam starch. Notably, elevated amylopectin levels in Chinese yam starch may hinder the interactions between starch and ligands during modification [15]. Hence, the influence of amylopectin content on the effectiveness of starch modification should be carefully considered.

Currently, the processes regulating the digestive and rheological properties of the Chinese yam starch–chlorogenic acid complex have not been fully explored. In this study, the Chinese yam starch–chlorogenic acid complexes were prepared via atmospheric pressure heating. We aimed to clarify how chlorogenic acid alters the multi-level structural features of modified Chinese yam starch and further modulate its digestibility and rheological behaviors. The influences exerted by chlorogenic acid upon the digestive performance and rheological behaviors of the Chinese yam starch–chlorogenic acid complex were characterized, and modern structural characterization techniques, including X-ray diffractometry (XRD), ^13^C CP/MAS nuclear magnetic resonance (NMR) spectroscopy, Fourier-transform infrared (FT-IR) spectroscopy, Raman spectroscopy and microscopy, and scanning electron microscopy (SEM), were applied to characterize long-range crystalline structure, short-range molecular order, molecular interactions and granular morphology. This research may provide a theoretical basis to improve the applicability of Chinese yam starch and support the development of low-glycemic foods.

## 2. Materials and Methods

### 2.1. Materials

Chinese yam (*Dioscorea opposita* Thunb.) (food grade) was provided by Fujian Shange Agricultural Comprehensive Development Food Co., Ltd. (Quanzhou, China). Chlorogenic acid (CA, purity 98%) and pullulanase (PUL, 2000 U/mL, pH 4.5–6.0, liquid, BR) was purchased from Shanghai Macklin Biochemical Co., Ltd. (Shanghai, China). All chemicals used were of analytical grade.

### 2.2. Preparation of Chinese Yam Starch

Non-edible segments of fresh yam tubers were discarded, and the leftover tubers were cleaned, peeled, sliced into fragments, and blended with deionized water at a solid–liquid ratio of 1:3 (*w*/*v*) to produce fresh Chinese yam slurry via a high-speed homogenizer. The slurry was homogenized for 3 min at 8000 rpm to fully destroy yam cell walls and release starch granules. This fresh yam slurry underwent rinsing with twice its volume of deionized water and anhydrous ethanol to collect a clarified supernatant following static sedimentation for 6 h at 4 °C. The washing-settling cycle was repeated three times to remove soluble sugars, proteins and polyphenols. Solid precipitate harvested from this procedure was recovered as crude Chinese yam starch. This crude starch fraction was dehydrated in an oven (101-1AB electric blast drying oven, Taisite Instrument Co., Ltd., Tianjin, China) at 40 °C to a constant weight, gently ground, passed through a 100-mesh screen, and designated as CYS (69.72% ± 0.499% amylopectin content, 26.28% ± 0.911% amylose content).

### 2.3. Preparation of Chinese Yam Starch–Chlorogenic Acid Complex

In short, starch slurries were formulated by dispersing starch flour within deionized water at a solid–liquid ratio of 1:20 (g/mL), then undergoing pregelatinization inside a boiling water bath over 10 min. Once cooled down to ambient temperature, these slurries underwent enzymatic breakdown with pullulanase (PUL, 200 U/g) incubated at 55 °C for 40 min. This enzymatic reaction was halted by heating the hydrolyzed slurries in boiling water for 10 min to fully deactivate the enzyme. Thereafter, chlorogenic acid (CA) was incorporated into the pre-gelatinized Chinese yam starch matrix at mass ratios of 3%, 5%, and 7% (*w*/*w*), and the resulting mixtures were incubated at 100 °C for 30 min. After reaction completion, the samples were rinsed with absolute ethanol and centrifuged at 5000 r/min for 15 min. Finally, the precipitates were dried at 40 °C to a constant weight and sieved through a 100-mesh screen. The obtained sample was pre-gelatinized Chinese Yam Starch–Chlorogenic Acid Complex (PCYS-CAC). For comparison, pre-gelatinized Chinese yam starch (PCYS) was prepared via an identical protocol without CA supplementation. The CA-PCYS complexes with varying CA contents were denoted as PCYS-3%CAC, PCYS-5%CAC, and PCYS-7%CAC, respectively. The 3%, 5% and 7% represent the nominal addition ratio of chlorogenic acid at the beginning of complex preparation, not the content of chlorogenic acid finally bound in the starch matrix.

### 2.4. In Vitro Digestion

**Figure 1:**
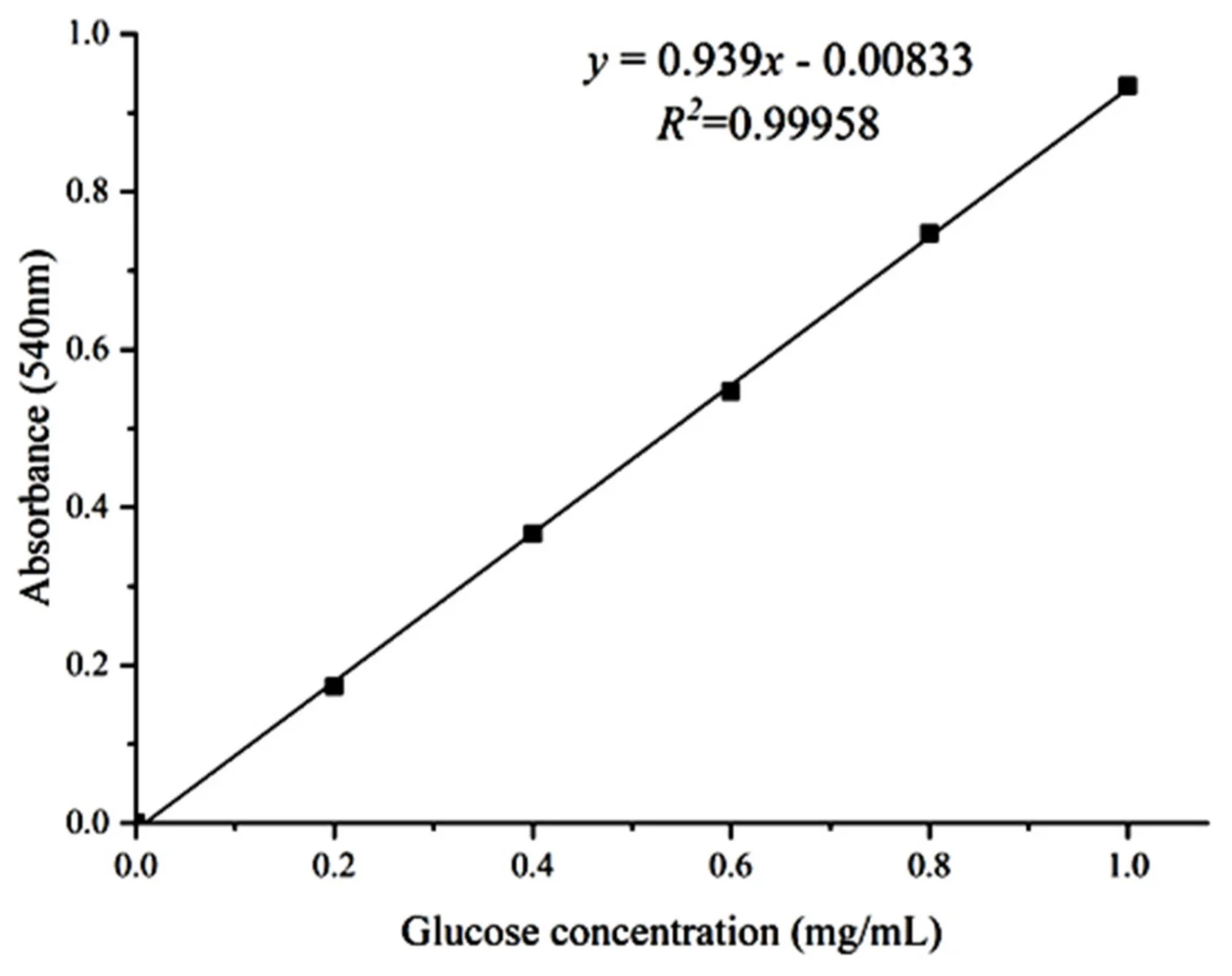
Standard curve of the glucose.

The glucose released from starch during hydrolysis was determined using the 3,5-dinitrosalicylic acid (DNS) color assay. Briefly, 300 mg starch sample (dry basis) was loaded into a dialysis bag pre-filled with phosphate buffer (20 mL, pH 5.2), α-amylase solution (10 mL, 260 U/mL) and glucoamylase solution (1 mL, 2500 U/mL). The sealed dialysis bag was immersed into a beaker containing phosphate buffer, and the enzymatic hydrolysis proceeded in a shaking water bath maintained at 37 °C and 150 rpm. At incubation intervals of 20 min and 120 min, 1 mL hydrolysate aliquots were withdrawn and immediately mixed with 3 mL 95% ethanol to terminate enzyme activity. The mixture underwent centrifugation at 3000 rpm for 10 min, then the resultant supernatant was harvested for measuring glucose concentration with the DNS assay by recording absorbance at 540 nm. Furthermore, the free glucose level within the specimen was computed based on the formula shown in Figure 1.

Rapidly digestible starch (RDS) and slowly digestible starch (SDS) were quantified following 20 min and 120 min incubation, separately. Resistant starch (RS) corresponds to non-hydrolyzed starch fraction retained upon 120 min of hydrolysis. Hydrolysis rate (*HR*), *RDS*, *SDS* and *RS* contents were calculated using Equations (1)–(5):

(1) $$G_{\left(t\right)}(mg)=\frac{G}{V}\times (331-V)+A-G_{\left(0\right)}$$

(2) $$HR(\%)=\frac{G(t)\times 0.9}{W}\times 100$$

(3) $$SDS(\%)=\frac{\left[G(120)-G(20)+G_{\left(0\right)}\right]\times 0.9}{W}\times 100$$

(4) $$RDS(\%)=\frac{G(20)\times 0.9}{W}\times 100$$

(5) RS(%)=100−RDS−SDS

where *G*_(_*_t_*_)_ = glucose mass (mg) released from starch after t min digestion; *G* = glucose mass obtained from standard curve (mg); *V* = volume of sampled hydrolysate (mL); 331 = total volume of digestion system (mL); *A* = glucose mass in sampled solution (mg); *G*_(0)_ = free glucose originally present in starch sample (mg); 0.9 = conversion factor for glucose to starch; *W* = mass of starch sample on dry basis (mg); *t* = incubation time (min).

### 2.5. Rheological Properties Measurements

The viscoelastic features of starch pastes were measured on a rotational rheometer (Physica MCR 301; Anton Paar, Co., Ltd., Graz, Austria) outfitted with a 25 mm parallel-plate fixture under a 1 mm gap separation. The system was kept at 25 °C for all measurements.

#### 2.5.1. Steady Shear Measurements

The samples were gelatinized with a Rapid Visco Analyzer (Perten Scientific Instruments Ltd., Stockholm, Sweden) and cooled to room temperature. The steady shear behavior was measured by linearly increasing the shear rate from 1.0 to 300 s^−1^ over 3 min, followed by an immediate linear decrease back to 1.0 s^−1^. Finally, the power-law relationship between the apparent viscosity and the shear rate was analyzed using the following equation.

(6) τ=K⋅γ⋅n

where *τ* is the shear stress (Pa), *γ* is the shear rate (s^−1^), *K* is the consistency index (Pa·s^n^), and *n* is the flow behavior index (dimensionless).

#### 2.5.2. Frequency Sweep Measurements

The frequency sweep test was run from 0.1 to 10 Hz within the linear strain range at 25 °C, and the mechanical spectra were recorded, which included the storage modulus (G′), loss modulus (G″), and the loss tangent (tanδ = G″/G′) as functions of frequency (Hz).

#### 2.5.3. Steady Shear Test: In-Shear Structural Regeneration

Structural regeneration capability of these specimens was characterized via the procedure described below. The shear rate was maintained at 1 s^−1^ for 180 s, increased to 300 s^−1^ for 60 s, and then returned to 1 s^−1^ for 240 s. The degree of structural recovery was calculated as the ratio of the average apparent viscosity during the last 180 s of the third stage to the average apparent viscosity during the last 150 s of the first stage.

### 2.6. Structural Characterization

#### 2.6.1. X-Ray Diffraction (XRD)

Crystallinity within starch particles was characterized via an X-ray diffractometer (Rigaku Corporation, Tokyo, Japan) fitted with a rotating copper anode X-ray tube. Measurement settings were listed below: Cu-Kα radiation was used as the target, with a scanning range of 3–45°, a scanning speed of 2°/min, and a step size of 0.02°. The relative crystallinity was calculated as the ratio of the crystalline diffraction peak area to the total diffraction area, using Origin Pro 8.5 software for background subtraction, partitioning of crystalline-amorphous regions and area integration.

#### 2.6.2. ^13^C CP/MAS Nuclear Magnetic Resonance (NMR)

High-resolution solid-state ^13^C CP/MAS NMR analysis of starch was performed using a 400 MHz spectrometer (Bruker 400M, Bruker Inc., Karlsruhe, Germany). The test was conducted at a frequency of 100.62 MHz, the rotation rate was 5 kHz, the single-spectrum scan accumulation was no less than 4000 times, the cycle delay was 2 s, and the acquisition time was 0.013 s

#### 2.6.3. Fourier-Transform Infrared (FT-IR) Spectral Measurement

FTIR vibrational signals for all specimens were acquired by means of an IRAffinity-1S spectrometer (Shimadzu Enterprise Management (China) Co., Ltd., Shanghai, China). All spectra were averaged from 25 scans over 400–4000 cm^−1^, with 4 cm^−1^ resolution at ambient temperature (approximately 25 °C) equipped with diffuse reflectance attachment. Starch specimens and KBr powder were combined at 1:100 *w*/*w* ratio, finely ground, and compressed into pellets before data collection. A background spectrum of pure KBr was acquired before the samples were scanned.

#### 2.6.4. Raman Microscopy

The Raman spectra of the samples were obtained using an InVia Raman confocal microscope (Horiba HR Evolution, Tokyo, Japan). The equipment parameters were as follows: spectral resolution, 4 cm^−1^; spectral scanning range, 2000–300 cm^−1^; laser wavelength, 780 nm; number of scans, 16; and exposure time, 10 s.

#### 2.6.5. Scanning Electron Microscopy (SEM)

The surface features of starch particles were characterized with magnifications of 1000× and 10,000× utilizing a scanning electron microscope (TESCAN MIRA4, TESCAN, Brno, Czech Republic). Sample powders were affixed to SEM stubs with double-sided tape. Excess loosely bound powder was blown away, and the residual samples underwent two gold sputter-coating runs. The whole measurement process was conducted under vacuum. The accelerating voltage was set to 3.0 kV for 1000× magnification, whereas 5.0 kV was employed to collect micrographs at 10,000×.

### 2.7. Statistical Analysis

Triplicate tests were arranged for all determination procedures, and obtained data were reported in the form of mean ± standard deviation. Plot generation for experimental data was completed in Origin Pro 8.5. SPSS software (Trial Version 18.0, SPSS Inc.) was applied to perform one-way analysis of variance and Duncan’s test. A significance threshold of *p* < 0.05 was set to judge the differences between groups.

## 3. Results and Discussion

### 3.1. In Vitro Digestive Properties

The digestion parameters of native and modified Chinese yam starches are shown in Table 1. The digestibility of PCYS at 20 and 120 min was slightly lower than that of CYS. This may be attributed to molecular rearrangement within the PCYS sample induced by the combined effect of pre-gelatinization and PUL treatment. According to a previous report, PUL treatment promotes the formation of a large number of long linear chains in starch, which can subsequently form a stable double helix structure after retrogradation, thereby enhancing resistance to enzyme digestion [16]. Compared with CYS, the digestibility of PCYS-3%CAC, PCYS-5%CAC, and PCYS-7%CAC decreased progressively. CA can bind to the active site of α-amylase, reducing the proportion of starch molecules available for enzyme hydrolysis [17], while the hydroxyl and phenolic groups of CA form strong hydrogen bonds with water molecules. This is believed to reduce the accessibility of water to digestive enzymes, further slowing the starch digestion rate. In addition, the RS content of PCYS-3%CAC, PCYS-5%CAC, and PCYS-7%CAC showed a significant increase compared with PCYS, and the RS content was positively correlated with the amount of CA added. This suggests that CA can dramatically promote the formation of RS in Chinese yam starch, consistent with previous research showing that CA can increase the RS content in wheat starch [18]. The hydrolysis rate of a starch is determined by its structural integrity, crystallinity, and amylose content. To better understand the mechanisms underlying the differences in the in vitro digestibility of the starch samples, their structural characteristics, including morphology, crystallinity, and long-/short-range molecular order, were further investigated.

### 3.2. Rheological Properties

#### 3.2.1. Steady Shear Behavior

**Figure 2:**
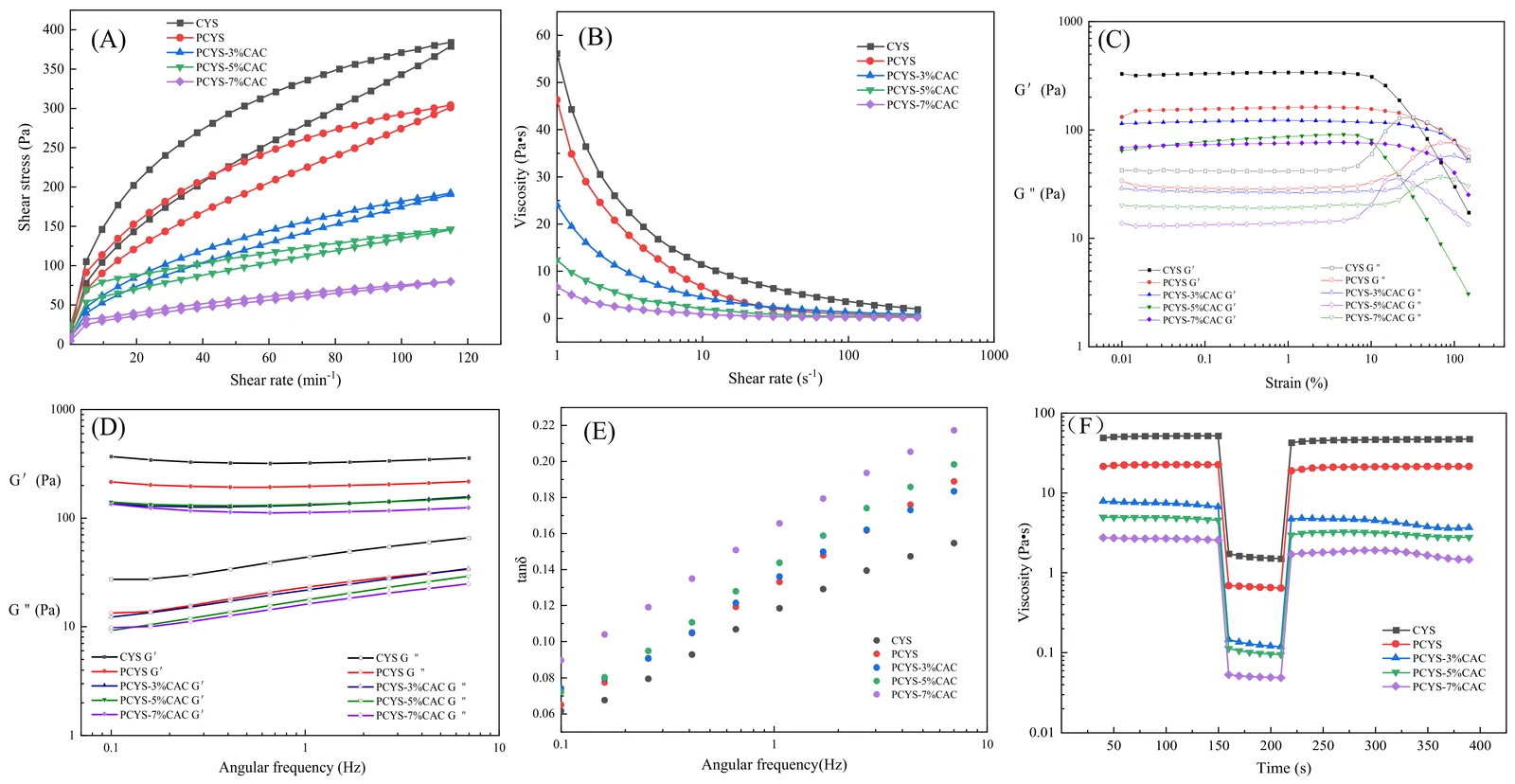
Rheological property curves of the native and modified Chinese yam starches: (**A**) flow curves, (**B**) viscosity curves, (**C**) strain sweep curves, (**D**) frequency sweep curves, (**E**) tanδ curves, and (**F**) in-shear structural recovery curves.

The trend of shear stress in the starch gel samples as a function of the shear rate is shown in Figure 2A. A large hysteresis loop area indicates that the starch sample exhibits a stronger thixotropic property. During shearing, CYS and modified Chinese yam starch gels displayed stress shear hysteresis, indicating their thixotropic behavior. CYS paste exhibited higher shear stress than other pastes under the same shear rate, likely due to strong interactions among starch molecules, leading to greater resistance to structural disruption. However, under enzymatic action, the interactions between starch molecules were weakened, resulting in a reduction in the shear stress of PCYS under the same conditions. The interactions between CA and starch also disrupted the entanglement and aggregation of starch molecules, further decreasing the shear stress of PCYS-3%CAC, PCYS-5%CAC, and PCYS-7%CAC compared with PCYS. This is consistent with the conclusions reported by Xu et al. (2021) [19].

The relationship between the apparent viscosity and shear rate for the starch gel samples is shown in Figure 2B. The apparent viscosity decreased sharply with increasing shear rate, indicating that all starch gel samples exhibited shear-thinning behavior, with the degree of shear-thinning enhanced by CA. Such a change originates from the breakage of starch chains induced by enzymes, which reduced aggregation and entanglement, loosened the network structure, and lowered the flow resistance. Moreover, the microcrystalline structures formed through the interactions between starch and CA reduced starch chain entanglement and destabilized the starch gel network, weakening its resistance to shear stress. This observation is inconsistent with the findings reported by Zeng et al. (2022) [20], who demonstrated that the formation of V-type complexes between starch and polyphenols further reduces the fluidity of starch gels. A plausible explanation is that the complexes in their study underwent extrusion treatment, which altered the rheological properties of the resultant complexes.

#### 3.2.2. Frequency Sweep

The strain scanning curves of the starch gels are shown in Figure 2C. The starch samples remained within the linear viscoelastic region at 1% strain, allowing frequency sweep measurements to be taken. The dynamic viscoelastic properties of starch paste were shown in Figure 2D. The storage modulus (G′) indicates the elasticity of starch gels, while the loss modulus (G″) reflects their viscosity. The G′ values of all starch pastes were higher than their G″ values, indicating that the samples exhibited solid-like behavior after cooling to 25 °C. For CYS, G′ was significantly greater than G″, indicating a strong gel structure. Compared with CYS, the G′ and G″ values of PCYS decreased markedly, likely due to the disruption of granule integrity and the ordered crystalline structure during enzymolysis treatment. This also indicates that enzymolysis treatment reduced the viscoelasticity of the starch gel. Additionally, the G′ and G″ values of PCYS-3%CAC, PCYS-5%CAC, and PCYS-7%CAC decreased slightly compared with PCYS, with the degree of decline increasing with CA concentration, consistent with the trends observed in pasting viscosities. The observed effect suggests that the interactions between starch and CA inhibit the expansion of starch granules, slowing the formation of gel connection regions between starch molecules [21]. Wang et al. [22] also reported that tea polyphenols can disrupt the compact physical structure of waxy maize starch, affecting its elasticity and viscosity.

The loss tangent (tan δ), defined as the ratio of G″ to G′, reflects the overall viscoelasticity of starch gels. An increase in tan δ indicates a reduction in the elasticity of the starch gel. The tan δ values of the starch samples are shown in Figure 2E. CYS exhibited the lowest tan δ value among all starch gel samples, indicating the formation of a strong gel network in the amorphous regions. The tan δ values of the five samples followed the order PCYS-7%CAC > PCYS-5%CAC > PCYS-3%CAC > PCYS > CYS, demonstrating that enzymolysis treatment and CA addition reduced the gel elasticity. Furthermore, CA further aggravated the decline in the elasticity of the starch gel, resulting in a looser gel structure at higher CA concentrations. This was consistent with the report by Wu et al., which showed that the addition of GTPs reduced the elasticity of rice starch gels [23].

#### 3.2.3. In-Shear Structural Recovery

The in-shear structural recovery curves of the starch gel samples are shown in Figure 2F. For CYS and PCYS, the viscosity showed a slight decrease before and after the change in shear rate. However, the three samples containing CA showed a significant decrease in viscosity. Enzyme treatment likely disrupted the original structure of the starch gel, and the addition of CA further intensified this effect. Therefore, the viscosity of the samples before and after the change in shear rate followed the order of CYS > PCYS > PCYS-3%CAC > PCYS-5%CAC > PCYS-7%CAC. The result is consistent with the findings of Liu et al., who reported that polysaccharides affected the recovery of the shear-induced internal structure of potato starch [24].

### 3.3. Structural Characteristics

#### 3.3.1. X-Ray Diffraction Analysis of Long-Range Ordered Structure

**Figure 3:**
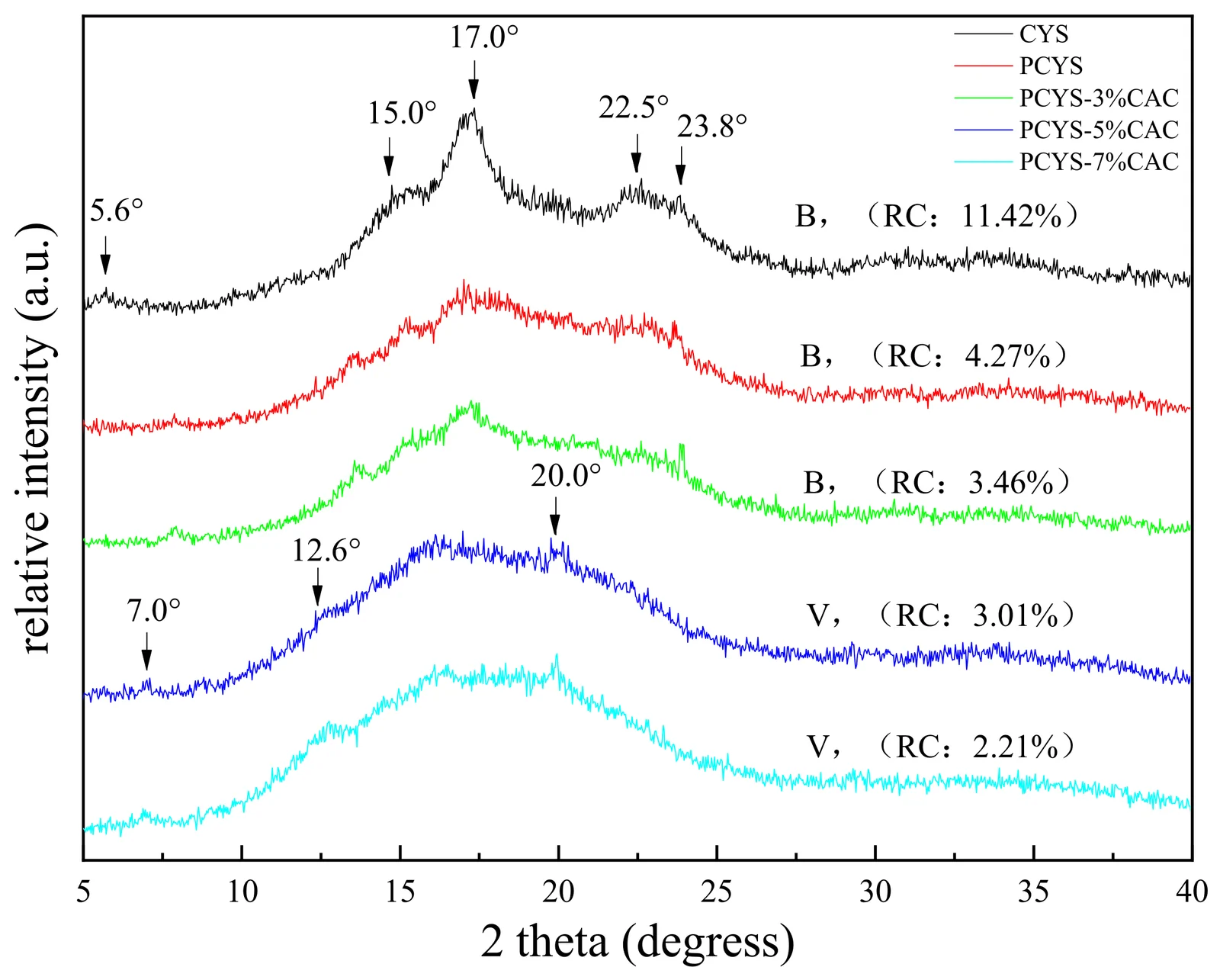
X-ray diffractograms of the native and modified Chinese yam starches. RC: relative crystallinity. B: B-type starch structure. V: V-type starch structure.

The XRD patterns of native and modified Chinese yam starches, along with their relative crystallinity, are shown in Figure 3. CYS, PCYS, and PCYS-3%CAC exhibited characteristic singlet peaks at 5.6°, 15.0°, and 17.0°, along with a bifurcated doublet peak at 22.5° and 23.8°, confirming a typical B-type starch structure. Although the diffraction intensity of these peaks for PCYS decreased substantially after pre-gelatinization, the positions of characteristic peaks remained unchanged. These peak positions confirm a typical B-type starch structure. This indicates that enzymolysis treatment or CA at low levels barely altered the native starch crystallinity. PCYS-5%CAC and PCYS-7%CAC exhibited sharp diffraction peaks at 7.0°, 12.6°, and 20.0°, characteristic of a typical V-type starch structure, indicating that CA interacted with the starch hydrophobic cavity to form inclusion complexes. The phenomenon may be attributed to PUL debranching, which promotes the release of linear starch chains from the interior of starch granules and generates additional sites for complex formation with guest molecules [25]. At the same time, higher CA concentrations may effectively inhibit the self-assembly and retrogradation of starch, resulting in the formation of V-type starch [26]. Additionally, the long-range order of each starch was characterized by its relative crystallinity. Compared with CYS, PCYS showed a significant decrease in relative crystallinity. This may be due to the high proportion of amylose produced during PUL debranching. Notably, the relative crystallinity of PCYS-3%CAC, PCYS-5%CAC, and PCYS-7%CAC decreased with increasing CA concentration, indicating that CA can disrupt the long-range ordered structure of starch, with the effect positively correlated to CA concentration.

#### 3.3.2. ^13^C CP/MAS NMR Spectroscopy Analysis of Short-Range Ordered Structure

**Figure 4:**
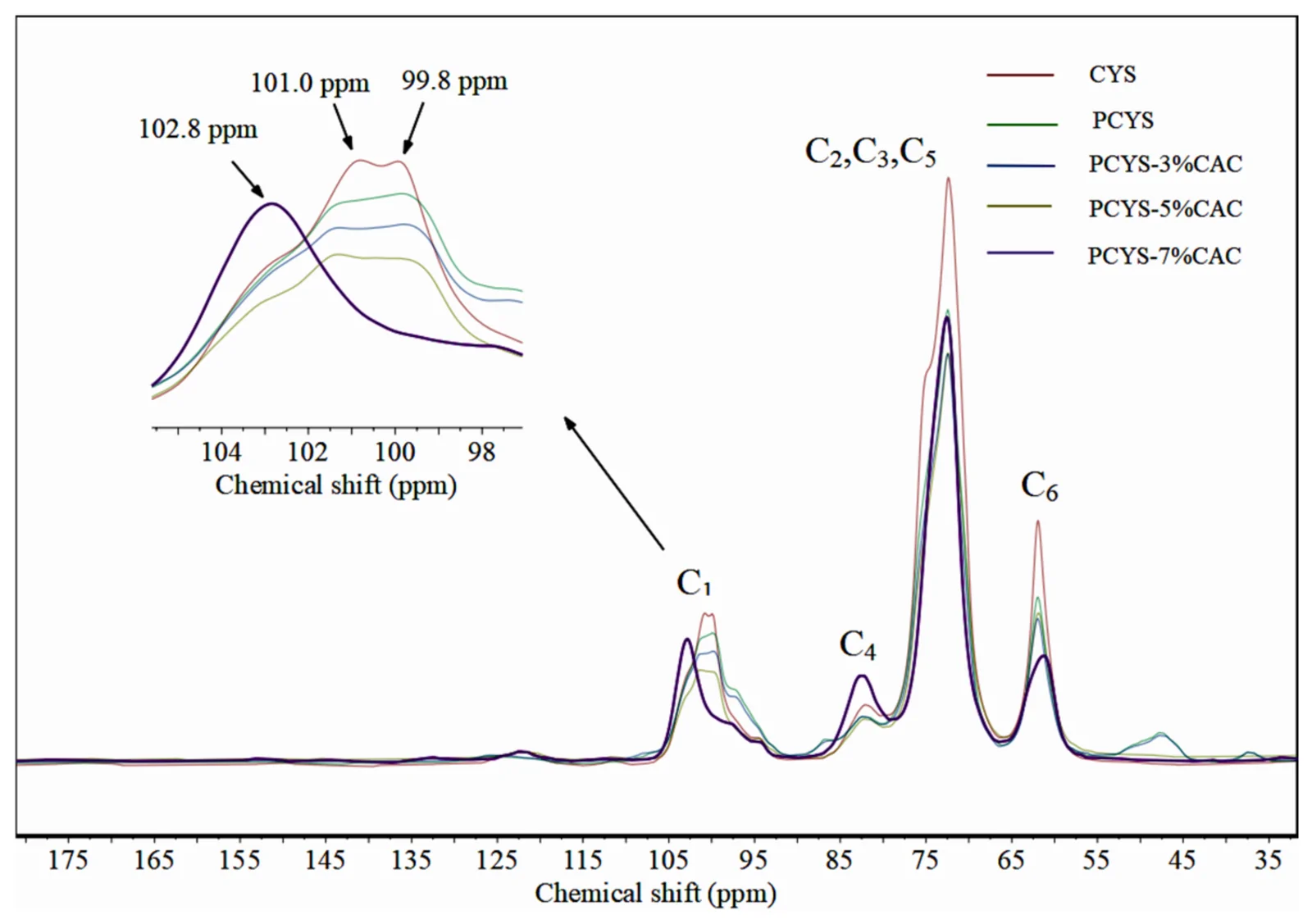
^13^C CP/MAS NMR spectra of the native and modified Chinese yam starches.

High-frequency ^13^C NMR spectroscopy was used to investigate the interactions between starch and small molecules, such as polyphenols, amino acids, and lipids. The ^13^C NMR chemical shifts of glucose carbon within the starch molecule fall into four main regions: 96–106 ppm (C_1_); 70–79 ppm (C_2_, C_3_, and C_5_); 80–84 ppm (C_4_); and 59–62 ppm (C_6_) [27]. The number of peaks in the C_1_ region reflects the starch crystal type, and the non-overlapping signals within 96–106 ppm can be used to quantify the single-helix (102–103 ppm) and double-helix (99–101 ppm) structures [28]. The peak area of C_4_ reflects the content of the amorphous structure. A broad, low-intensity peak at 82 ppm indicates that the starch has a relatively high degree of amorphous structure [29]. As shown in Figure 4, CYS, PCYS, PCYS-3%CAC, and PCYS-5%CAC displayed two types of chemical shifts in the C_1_ region, indicating that all four starch samples were B-type. In contrast, PCYS-7%CAC exhibited a single peak at 102.8 ppm, which is characteristic of V-type starch [30]. This discrepancy with the XRD results may arise from the differences in the detection focus and precision of the two methods. NMR is mainly used to analyze the short-range order of starch molecules, while XRD focuses on the long-range ordered structure [31]. As shown in Table 2, the relative crystallinities of CYS, PCYS, PCYS-3%CAC, PCYS-5%CAC, and PCYS-7%CAC decreased gradually (20.27%, 16.29%, 16.08%, 15.61%, and 14.52%, respectively), which is consistent with the XRD results. Compared with CYS, PCYS showed a decrease in double-helix and amorphous content, whereas the single-helix content increased. This may be due to the combined effects of pregelatinization and PUL treatment, which cause the double-helix structure in PCYS to unwind and correspondingly affect the single-helix and amorphous content [32]. Furthermore, with increasing CA concentration, the double-helix content in PCYS-3%CAC, PCYS-5%CAC, and PCYS-7%CAC decreased significantly, while the contents of amorphous and single-helix structures increased. The pronounced decrease in double-helix structures in these samples may be attributed to deeper starch gelatinization and the combined effect of CA. Liu et al. [33] similarly reported that higher gallic acid concentrations led to a rise in the single-helix content in the rice starch–gallic acid complex. Furthermore, Cao et al. [34] reported that phenolic compounds with smaller steric hindrance can form single-helix structures with starch and further induce the formation of double-helix arrangements. It is worth noting that the benzene ring in CA creates considerable steric hindrance, thereby inhibiting the formation of double-helix structures in starch. Therefore, in the PCYS-7%CAC sample, the single-helix content was the highest, while the double-helix content was the lowest.

#### 3.3.3. FT-IR and Raman Spectroscopy

**Figure 5:**
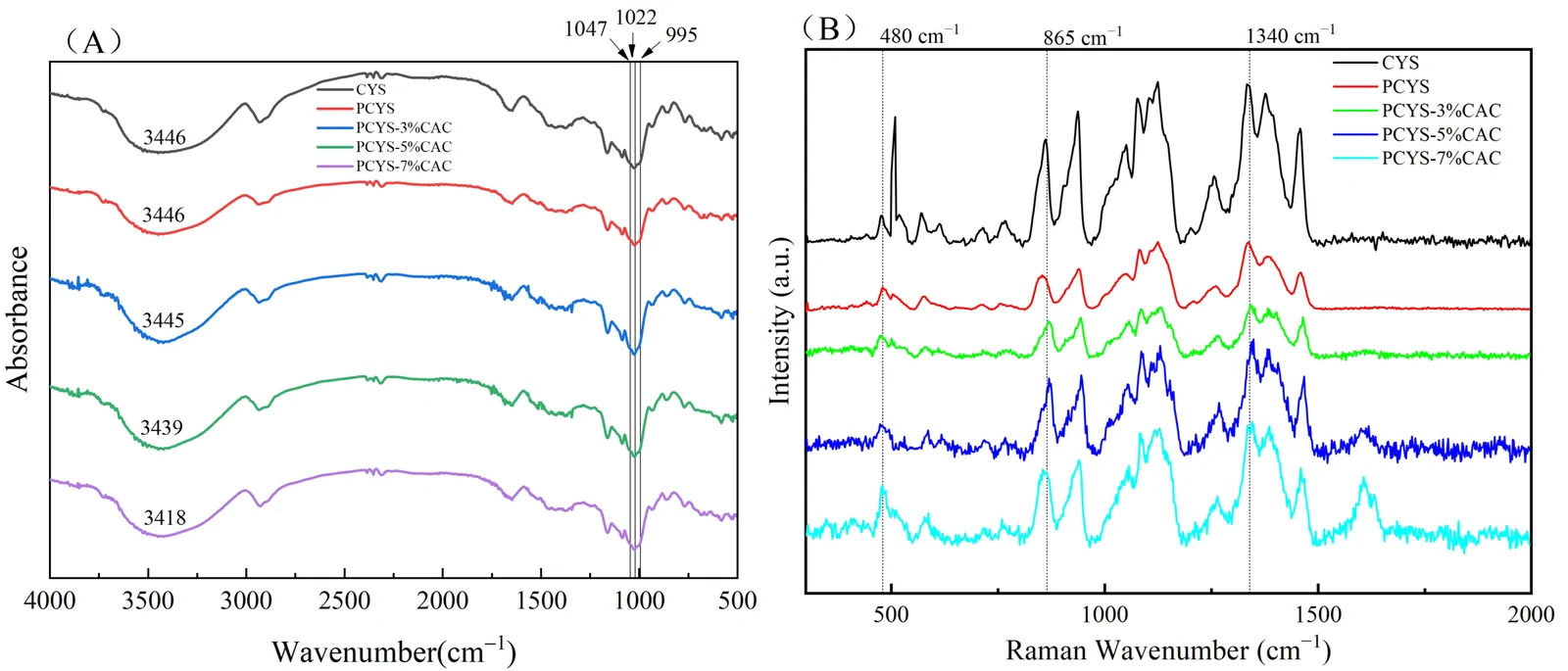
FTIR (**A**) and Raman spectra (**B**) of native and modified Chinese yam starches.

The FT-IR spectra of native and modified Chinese yam starches are shown in Figure 5A. No new peaks were observed in any of the modified starches, suggesting that the interactions between starch and CA are primarily non-covalent. The bands in the 3000–3600 cm^−1^ region were attributed to the stretching vibration of hydroxyl groups, and the shift of these bands toward lower wavenumbers indicates strengthened intermolecular hydrogen bonding []. Compared with CYS, the 3000–3600 cm^−1^ spectral region of PCYS remained unchanged, indicating that pre-gelatinization did not affect the number of hydrogen bonds in starch molecules. The absorption peaks of PCYS-CAC samples in the 3000–3600 cm^−1^ region shifted to lower wavenumbers with increasing CA content, suggesting the formation and strengthening of hydrogen bonds between starch and CA [35].

The bands at 1047 and 1022 cm^−1^ correspond to the crystalline and amorphous regions of the starch sample, respectively. Thus, the 1047/1022 cm^−1^ absorbance ratio (*R*_1047/1022_) can be used to assess the degree of short-range order in starch samples. The 1022/995 cm^−1^ absorbance ratio (*R*_1022/995)_ provides a measure of the double-helix content in starch molecules [36]. The *R*_1047/1022_ and *R*_1022/995_ values were lower in PCYS than in CYS (Table 3). This may be attributed to enzymolysis treatment, which breaks hydrogen bonds within starch molecules and further disrupts the short-range order and double-helix structure. Notably, *R*_1047/1022_ values increased after complexation with CA, indicating that CA increased the short-range order of starch. In addition, *R*_1022/995_ decreased, indicating that CA disrupted the starch double-helix structure.

Raman spectroscopy is more sensitive to the fundamental and symmetrical vibrations of polar molecules and bonds with low polarity [37]. Therefore, Raman spectroscopy can serve as a complementary tool for analyzing the weak bands observed in FT-IR and can also provide insights into the crystallization and structural characteristics of starch [38]. The stretching vibration bands at 865 and 1340 cm^−1^ were assigned to C_1_–O–C_4_ glucosyl ring vibrations and to variations in the C–OH bond angle, respectively. These vibrations are, respectively, associated with the double-helix and single-helix structures within the straight-chain starch []. In addition, the full width at half maximum (FWHM) of the 480 cm^−1^ band can be used to evaluate the short-range order of crystalline starch, as it is sensitive to the molecular arrangement of the starch chains. A lower FWHM value indicates a higher degree of short-range order in the molecular arrangement of the starch sample [39]. As shown in Figure 5B and Table 3, compared with CYS, the intensity of several diffraction peaks in PCYS decreased, and the FWHM at 480 cm^−1^ for the PCYS sample was significantly higher than that of the CYS sample. Under the action of PUL, the chemical bonds between PCYS molecules were disrupted, increasing the disorder of the starch molecules, which is consistent with the findings of Wang et al. [40]. However, the FWHM of PCYS-3%CAC, PCYS-5%CAC, and PCYS-7%CAC were notably lower than that of PCYS, while the intensity of several diffraction peaks in these samples showed a gradual increase, indicating that the integration of CA significantly enhanced the short-range molecular order of starch. This enhancement can be attributed to the interaction between CA and amylose, which is consistent with the FT-IR findings. Meng et al. and Yu et al. also reported that gallic acid and resveratrol could enhance the short-range order of starch [41,42].

**Figure 6:**
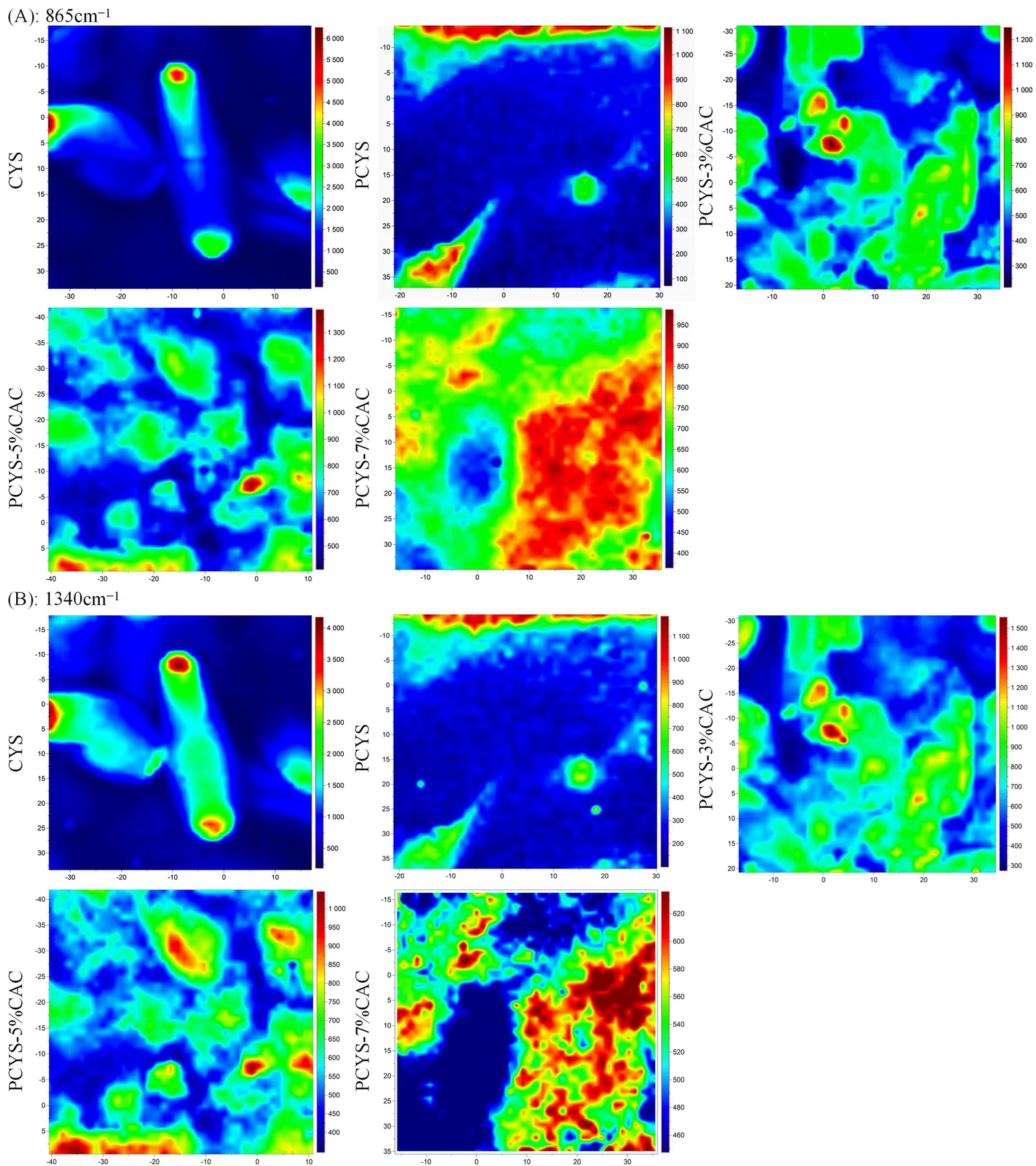
Raman maps at 865 cm^−1^ (**A**) and 1340 cm^−1^ (**B**) of native and modified Chinese yam starches.

Raman imaging spectroscopy can more intuitively visualize the distribution of double-helix retrograded structures (865 cm^−1^) and single-helix structures (1340 cm^−1^) in amylose. Furthermore, the images use a red–yellow–green–blue gradient, where red represents the highest content and blue represents the lowest content. As shown in Figure 6, red regions at 865 and 1340 cm^−1^ are more pronounced in PCYS than in CYS, indicating that pre-hydrolysis and PUL treatment enhanced the double-helix retrograded structure and single-helix structure of the amylose in the sample. Similarly, CA enhanced the double- and single-helix structure in amylose. Therefore, in the PCYS-3%CAC, PCYS-5%CAC, and PCYS-7%CAC samples, the red regions increased, while the blue regions decreased, and this effect became more pronounced with higher CA concentrations. This is consistent with the findings of Su et al., who reported that laminarin could enhance the formation of double- and single-helix structures in lotus seed amylose [43].

#### 3.3.4. Morphological Properties

**Figure 7:**
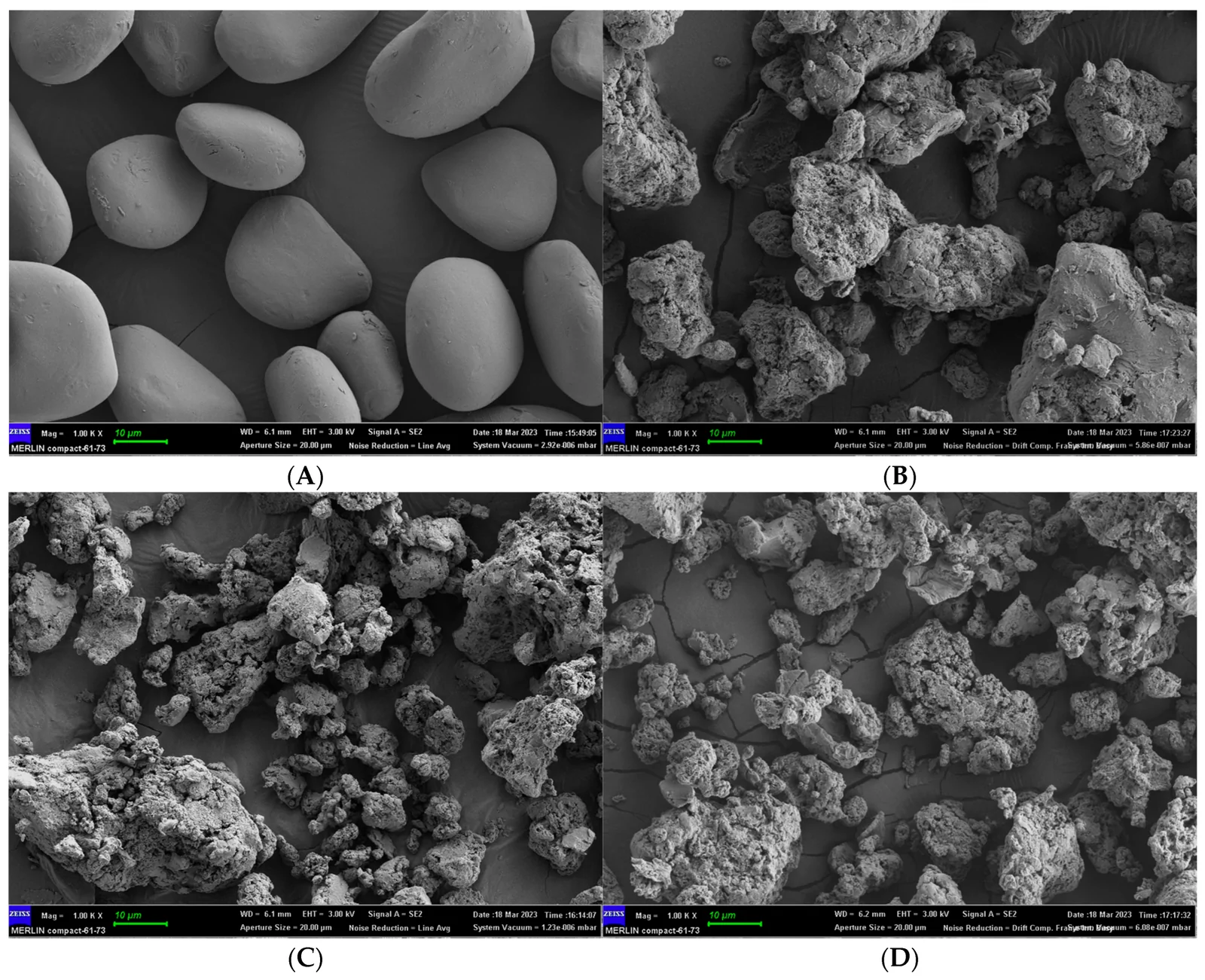

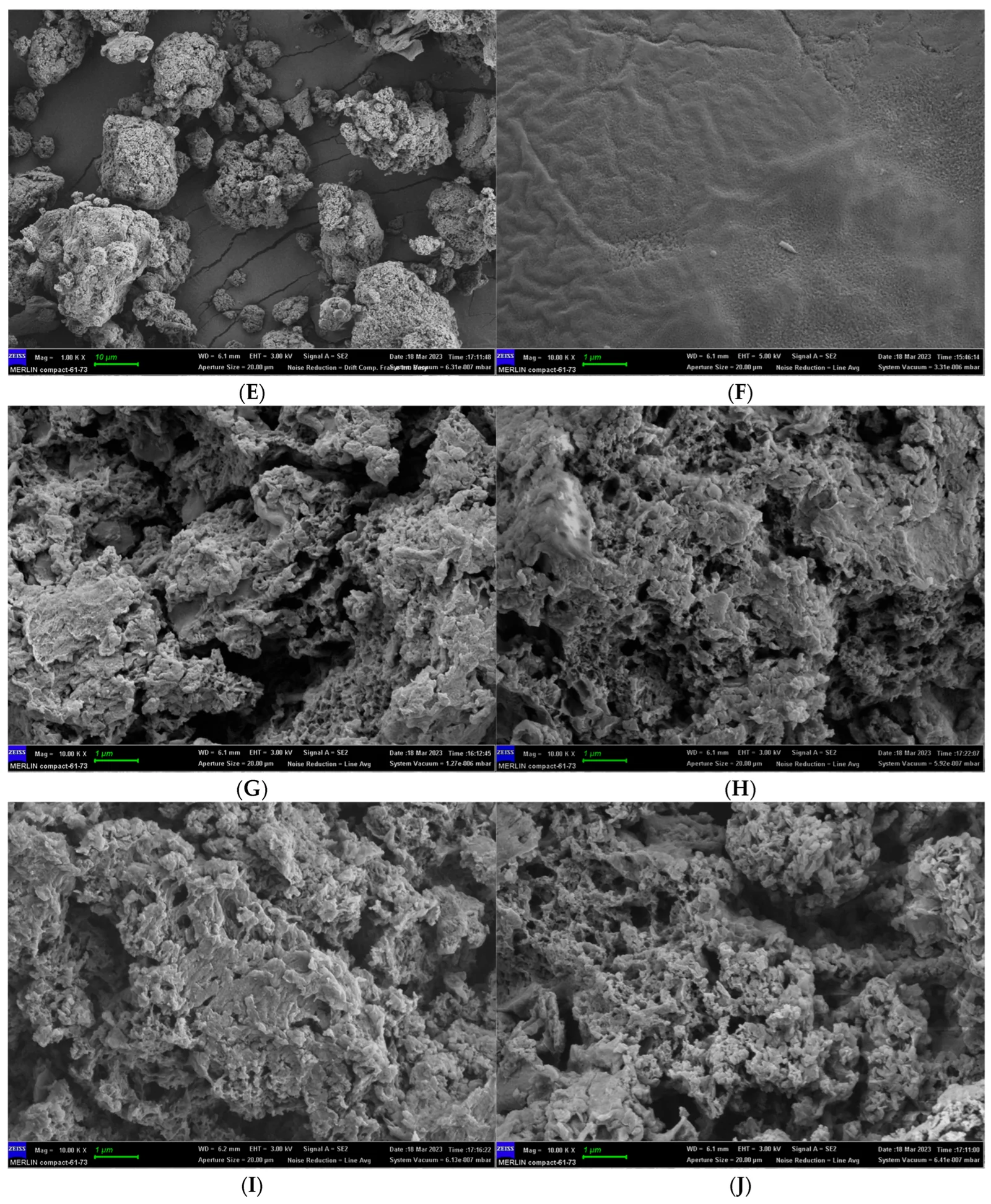
Scanning Electron Microscopy (SEM) images of native and modified Chinese yam starches at 1000×: (**A**) CYS, (**B**) PCYS, (**C**) PCYS-3%CAC, (**D**) PCYS-5%CAC, and (**E**) PCYS-7%CAC; at 10,000×: (**F**) CYS, (**G**) PCYS, (**H**) PCYS-3%CAC, (**I**) PCYS-5%CAC, and (**J**) PCYS-7%CAC.

The morphologies of native and modified Chinese yam starch granules were observed by SEM (Figure 7). The CYS granules (Figure 7A) exhibited an irregular oval shape with a smooth surface and flat edges. Compared with CYS, the surface of PCYS granules (Figure 7B) appeared concave, with aggregation and voids observed after enzymatic treatment. This can be attributed to the swelling of starch granules and the leaching of amylose [44]. The surfaces of all modified starch granules exhibited irregular shapes, rough textures, and prominent pores. Moreover, the size and shape of pores in PCYS, PCYS-3%CAC, PCYS-5%CAC, and PCYS-7%CAC significantly differed (Figure 7B–E). Specifically, the granule surfaces of PCYS-3%CAC, PCYS-5%CAC, and PCYS-7%CAC exhibited looser, more porous, and denser network structures, accompanied by a gradual reduction in pore size and wall thickness (Figure 7H–J). This phenomenon can be attributed to CA accelerating the dissociation of the original starch structure, with the degree of damage positively correlated with the amount of CA added [32]. The result is consistent with a previous study showing that the gel network structure of rice starch can be disrupted by the addition of tea polyphenols [45].

## 4. Conclusions

This study systematically investigated how chlorogenic acid (CA) reorganizes the structure of pregelatinized Chinese yam starch (CYS) and modulates its rheological properties and in vitro digestibility. Pregelatinization disrupted the native ordered structure of CYS, while the introduction of CA further altered starch molecular packing. The addition of CA induced a looser porous matrix and increased the short-range order of starch. Such structural rearrangement reduced the shear stress, swelling degree, gel strength and viscosity of starch complexes and weakened the shear-thinning behavior. Meanwhile, CA reduced starch crystallinity and facilitated the formation of V-type crystalline structure in PCYS-7%CA. Benefiting from these structural modifications, PCYS-5%CA and PCYS-7%CA effectively retarded starch digestion and raised the resistant starch (RS) content in a CA-concentration-dependent manner. Taken together, this work reveals the structure–rheology–digestibility relationship of the CYS–CA complex, demonstrating that CA can act as a promising functional ingredient to slow starch digestion in starchy foods. Further research is still required to explore the practical application of the CYS–CA complex in real food systems.

## Figures and Tables

**Table 1:** In vitro digestive properties of native and modified Chinese yam starches.

Samples	*HR* (%)		*RDS* (%)	*SDS* (%)	*RS* (%)
	20 min	120 min			
CYS	31.35 ± 0.34 ^ab^	57.95 ± 0.31 ^b^	28.22 ± 0.31 ^ab^	29.72 ± 0.50 ^b^	42.06 ± 0.31 ^b^
PCYS	30.04 ± 0.24 ^b^	61.90 ± 3.15 ^a^	27.03 ± 0.22 ^b^	34.86 ± 3.00 ^a^	38.10 ± 3.15 ^c^
PCYS-3%CAC	33.81 ± 3.09 ^a^	60.56 ± 0.17 ^a^	30.43 ± 2.78 ^a^	30.13 ± 2.80 ^b^	39.44 ± 0.17 ^c^
PCYS-5%CAC	26.98 ± 0.82 ^c^	55.81 ± 0.20 ^b^	24.28 ± 0.74 ^c^	31.52 ± 0.93 ^ab^	44.19 ± 0.20 ^b^
PCYS-7%CAC	24.89 ± 0.54 ^c^	50.36 ± 0.19 ^c^	22.40 ± 0.48 ^c^	27.96 ± 0.29 ^b^	49.64 ± 0.19 ^a^

^a–c^ Means with different letters in the same column are significantly different (*p* < 0.05). Values are expressed as means ± standard deviation (SD), *n* = 3.

**Table 2:** The amorphous structure content, double and single helix contents, and relative crystallinity of the native and modified Chinese yam starches.

Samples	Amorphous (%)	Double Helix (%)	Single Helix (%)	Relative Crystallinity (%)
CYS	29.17 ± 0.060 ^b^	55.24 ± 0.17 ^a^	15.83 ± 0.63 ^e^	20.26 ± 0.11 ^a^
PCYS	21.26 ± 0.27 ^d^	54.22 ± 0.12 ^b^	24.95 ± 0.75 ^d^	16.29 ± 0.085 ^b^
PCYS-3%CAC	20.72 ± 0.48 ^d^	52.86 ± 0.26 ^c^	26.21 ± 0.90 ^c^	16.06 ± 0.12 ^c^
PCYS-5%CAC	25.01 ± 0.27 ^c^	41.72 ± 0.17 ^d^	33.23 ± 0.53 ^b^	15.63 ± 0.092 ^d^
PCYS-7%CAC	30.49 ± 0.29 ^a^	34.43 ± 0.32 ^e^	35.43 ± 0.34 ^a^	14.54 ± 0.097 ^e^

^a–e^ Means with different letters in the same column are significantly different (*p* < 0.05). Values are expressed as means ± standard deviation (SD), *n* = 3.

**Table 3:** The short-range ordered structure and FWHM of the Raman band at 480 cm^−1^ of native and modified Chinese yam starches.

Samples	995 cm^−1^	1022 cm^−1^	1047 cm^−1^	*R*_1047/1022_	*R*_1022/995_	FWHM at 480 cm^−1^
CYS	39.1034 ± 0.1988 ^e^	36.8265 ± 0.03684 ^e^	39.8914 ± 0.03685 ^e^	1.0832 ± 0.002574 ^a^	0.9418 ± 0.002574 ^a^	16.6956 ± 0.8658 ^c^
PCYS	57.9098 ± 0.1683 ^a^	53.9340 ± 0.03418 ^a^	57.3608 ± 0.03418 ^a^	1.0635 ± 0.003068 ^c^	0.9313 ± 0.003068 ^b^	24.6197 ± 0.3396 ^a^
PCYS-3%CAC	55.3761 ± 0.04948 ^b^	51.1383 ± 0.03418 ^b^	54.9155 ± 0.06144 ^b^	1.0739 ± 0.001033 ^b^	0.9235 ± 0.001033 ^c^	20.8614 ± 0.4430 ^b^
PCYS-5%CAC	46.3200 ± 0.04972 ^c^	42.5273 ± 0.06465 ^c^	46.2113 ± 0.06465 ^c^	1.0866 ± 0.00372 ^a^	0.9181 ± 0.003728 ^d^	16.3133 ± 0.3617 ^c^
PCYS-7%CAC	45.2772 ± 0.05407 ^d^	41.2349 ± 0.09106 ^d^	44.8627 ± 0.09106 ^d^	1.0880 ± 0.005348 ^a^	0.9107 ± 0.005348 ^e^	14.6693 ± 0.5178 ^d^

^a–e^ Means with different letters in the same column are significantly different (*p* < 0.05). Values are expressed as means ± standard deviation (SD), *n* = 3.

## Data Availability

The original contributions presented in this study are included in the article. Further inquiries can be directed to the corresponding author.
